# Plasma Metabolic Profiling Analysis of Gout Party on Acute Gout Arthritis Rats Based on UHPLC–Q–TOF/MS Combined with Multivariate Statistical Analysis

**DOI:** 10.3390/ijms20225753

**Published:** 2019-11-15

**Authors:** Yuming Wang, Chenghao Bi, Wentao Pang, Yuechen Liu, Yu Yuan, Huan Zhao, Tianpu Zhang, Yungang Zhao, Yubo Li

**Affiliations:** 1 Tianjin University of Traditional Chinese Medicine, No. 10, Poyang Lake Road, West Zone, Tuanbo New City, Jinghai District, Tianjin 300193, China; wangyuming226@163.com (Y.W.); bch5039312@163.com (C.B.); liuyuechen777@163.com (Y.L.); yuanyu19980717@163.com (Y.Y.); m15612484815@163.com (H.Z.); z1179721543@163.com (T.Z.); 2 Tianjin Key Laboratory of Exercise Physiology and Sports Medicine, Institute of Sports and Health, Tianjin Sport University, No. 16, Donghai Road, Tianjin 300193, China; pangwentaoyx@163.com

**Keywords:** Gout Party, Metabolomics, Gout, Acute Gouty Arthritis, UHPLC–Q–TOF/MS, Biomarkers

## Abstract

Gout Party is a Chinese medicine prescription composed of *Aconiti Lateralis Radix Praeparaia, Aconiti Radix Cocta, Cremastrae Pseudobulbus Pleiones Pseudobulbus, Smilacis Glabrae Rhizoma, Rehmanniae Radix*, and *Glycyrrhizae Radix et Rhizoma*, which can relieve joint pain caused by gouty arthritis (GA) and rheumatoid, and has a therapeutic effect on acute gouty arthritis (AGA). However, little information is available on the molecular biological basis and therapeutic mechanism of Gout Party for the treatment of AGA. AGA model was established by injecting sodium urate, and colchicine served as a positive control drug. We established a metabolomic method based on ultra-high-performance liquid chromatography–tandem quadrupole/time-of-flight mass spectrometry (UHPLC–Q–TOF/MS) to analyze the plasma samples of model group rats and blank group rats. Multiple statistical analyses, including principal component analysis (PCA) and partial least square discrimination analysis (PLS-DA), were used to examine metabolite profile changes in plasma samples. Finally, we identified 2–ketobutyric acid, 3–hexenedioic acid, but–2–enoic acid, and so on; 22 endogenous metabolites associated with AGA. After successful molding, we found that 2–ketobutyric acid, 3–hexenedioic acid, but–2–enoic acid, argininic acid, galactonic acid, lactic acid, equol 4′–O–glucuronide, deoxycholic acid glycine conjugate, glycocholic acid, sphinganine 1–phosphate, LPE (0:0/20:3), LPE (0:0/16:0), LPC (15:0) decreased significantly (*p* < 0.05 or *p* < 0.01), alanine, erythrulose, 3–dehydrocarnitine, m–methylhippuric acid, 3–hydroxyoctanoic acid, p–cresol sulfate, estriol 3–sulfate 16–glucuronide, 10–hydroxy–9–(phosphonooxy)octadecenoate, docosahexaenoic acid increased significantly (*p* < 0.05 or *p* < 0.01). After Gout Party treatment, 14 biomarkers had a tendency to normal conditions. These above biomarkers were mainly involved in fatty acid metabolism, bile acid metabolism, amino acid metabolism, and energy metabolism pathways. These results suggested that Gout Party exerted therapeutic effects of treating AGA by improving energy metabolism disorder and amino acid metabolism dysfunction, and attenuating fatty acid metabolism abnormal and inflammation. The results of this experiment provided a reference for revealing the metabolic mechanism produced by Gout Party in the treatment of AGA, but the subsequent studies need to be further improved and supported by relevant cell experiments and clinical experiments.

## 1. Introduction

Gout is an inflammatory arthritis caused by the deposition of sodium urate crystals (SUCs) in joints and tissues [1]. Hyperuricemia is a clinical manifestation of gout, and severe renal dysfunction decreases the ability of the kidneys to excrete uric acid. AGA is an important complication of gout [2]. Hyperuricemia is caused by abnormally high levels of uric acid in the blood, and it has been recognized as a key factor in the development of gout [3,4]. The incidence of gout increases with increasing serum uric acid (SUA) levels [5,6]. Sodium urate deposition in the joint is an important cause of acute gouty arthritis [7]. In addition, gout is also closely related to the occurrence of metabolic syndrome, kidney damage, and cardiovascular disease [8,9,10]. Although progress has been made in recent years in uncovering the pathogenesis of GA, the prevalence of GA has intensified in recent decades with lifestyle changes [11], and the incidence of AGA has also increased. Therefore, accurate, rapid, and reliable diagnostic methods and treatments are needed for these conditions. Some progress has been made in the treatment of AGA with the development of the traditional Chinese medicine (TCM) Gout Party; however, the mechanism of action of the drug in the prevention and treatment of AGA has been rarely studied from the perspective of metabolomics; thus, studying the regulation of endogenous metabolism by Gout Party and the mechanism of action of the treatment will be of great significance in the clinical treatment of AGA.

The most commonly used drugs for treating gout are anti–inflammatory (colchicine and indomethacin) and uric acid–lowering drugs (allopurinol and benzbromarone). In modern Chinese medicine, Chinese herbal medicines or extracts have been increasingly used for treating gout and AGA [12,13]. Moreover, Gout Party is an effective treatment for gout and AGA based on the TCM syndrome differentiation theory. Gout Party is a Chinese medicine that was authorized after long–term clinical practice research. Its Chinese name is *Tongfengfang*, and it is derived from traditional Chinese medicine prescription Tongfengding capsules. Its components are *Aconiti Lateralis Radix Praeparaia* (hēi shun piàn), *Aconiti Radix Cocta* (chuān wū), *Cremastrae Pseudobulbus Pleiones Pseudobulbus* (shān cí gū), *Smilacis Glabrae Rhizoma* (tǔ fú líng), *Rehmanniae Radix* (dì huáng), and *Glycyrrhizae Radix et Rhizoma* (gān cǎo). According to the concept of Chinese medicine, Gout Party had the effects of clearing heat, dampness, detoxification, promoting blood circulation, reducing inflammation, hurricane, and relieving pain. The composition of many traditional Chinese medicine prescriptions is to some extent the same. For example, Chen [14] verified the effect of Quzhuotongbi Decoction by rat model of gout induced by sodium urate. Studies revealed that sodium urate accelerated the secretion of interleukin (IL)–6 and IL–1β, causing inflammatory effects on the joints [15,16].

Metabolomics is an emerging and rapidly evolving discipline that can describe biomarkers or characterize disease by detecting, identifying, and quantifying small–molecule endogenous metabolites in biological samples such as plasma and urine. In recent years, metabolomic methods have been successfully used to identify abnormal early signals, biomarkers, and biological pathway characteristics, and facilitate disease diagnoses [17,18,19]. Metabolomics has unique advantages in explaining the pharmacological mechanisms of drugs via its multi-channel holistic research model, which provides new ideas and methods for studying the holistic mechanisms of TCMs [20]. UHPLC–Q–TOF/MS is a powerful tool for metabolomics research that is extremely useful for research involving the mechanism of action of Chinese herbal medicine products [21]. Through the metabolomic analysis of endogenous metabolites, the pathogenesis of AGA can be explored, and a reliable basis for disease prevention, detection, diagnosis, and treatment can be provided. Through the metabolomic study of Gout Party, we preliminarily revealed its effects and treatment mechanism.

Rat models are very valuable in the study of gout and gouty arthritis [14]. Our study was based on UHPLC–Q–TOF/MS technology, in which the plasma of AGA model rats is used. Identification of potential biomarkers via multivariate analysis methods such as PCA and PLS–DA were performed to elucidate the pathogenesis of AGA and explore the mechanism of Gout Party in treating AGA. This strategy represented a reliable local method for further studying AGA. Simultaneously, it combined metabolomics with a TCM prescription to provide a foundation for studying the influence of TCMs in the treatment of AGA.

## 2. Results

### 2.1. Histopathological Evaluation

**Figure 1:**
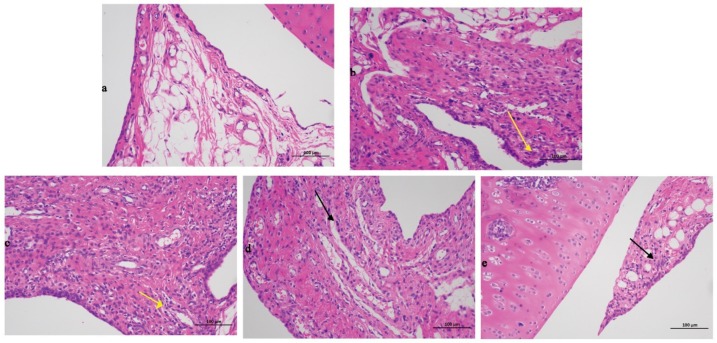
Histopathological evaluation of different treated groups. (**a**) Blank group. (**b**) Model group. (**c**) Positive control group. (**d**) High dose group. (**e**) Low dose group. The black and yellow arrows pointed to inflammatory cells.

The findings for the tibial tissue sections were presented in Figure 1. Compared with the blank group data, the model group exhibited a significant severe inflammatory reaction with a large number of immersed inflammatory cells (yellow arrow in Figure 1b), illustrating that the rats AGA model was successfully established. Compared with the model group results, the positive control group was also typified by inflammatory cell infiltration (yellow arrow in Figure 1c). The high-dose (black arrow in Figure 1d) and low-dose groups (black arrow in Figure 1e) exhibited significantly less inflammatory cell infiltration, demonstrating that Gout Party can significantly reduce synovial tissue inflammation in rats with AGA.

### 2.2. Determination of IL-1β and IL-6 in Plasma.

**Figure 2:**
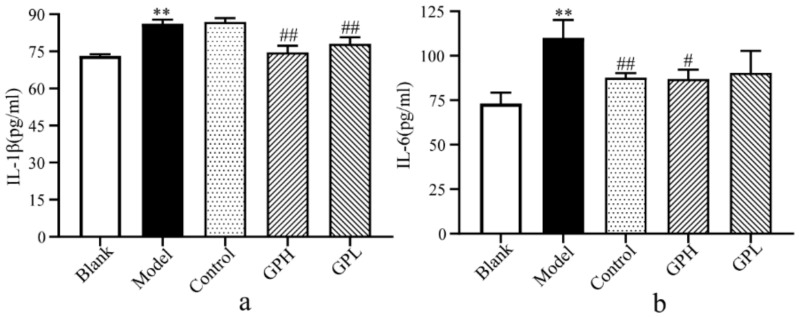
The results of biochemical indicators. (**a**) Changes of IL–1β in plasma of different groups. (**b**) Changes of IL–6 in plasma of different groups. (**: Significantly increased compared with blank group (*p* < 0.01); #: Significantly decreased compared with model group (*p* < 0.05); ##: Extremely significantly decreased compared with model group (*p* < 0.01).

In this experiment, we used ELISA to detect IL-6 and IL-1β levels in five groups (Figure 2). After successful modeling, IL-1β and IL-6 levels significantly increased in the model group compared with the blank group (*p* < 0.01). Compared with the model group after 7 days of administration, the IL-1β in the high-dose group and the low-dose group were significantly lower than those in the model group (*p* < 0.01). The level of IL-6 in the positive control group was significantly lower than that in the model group (*p* < 0.01). At the same time, we found a significant decrease in IL-6 in the high-dose group (*p* < 0.05). The results suggested that Gout Party can reduce the levels of IL-6 and IL-1β during inflammation.

### 2.3. Chemical Characterization of Gout Party

We tested instrument precision, method repeatability, and sample stability in accordance with traditional law enforcement methods.

**Figure 3:**
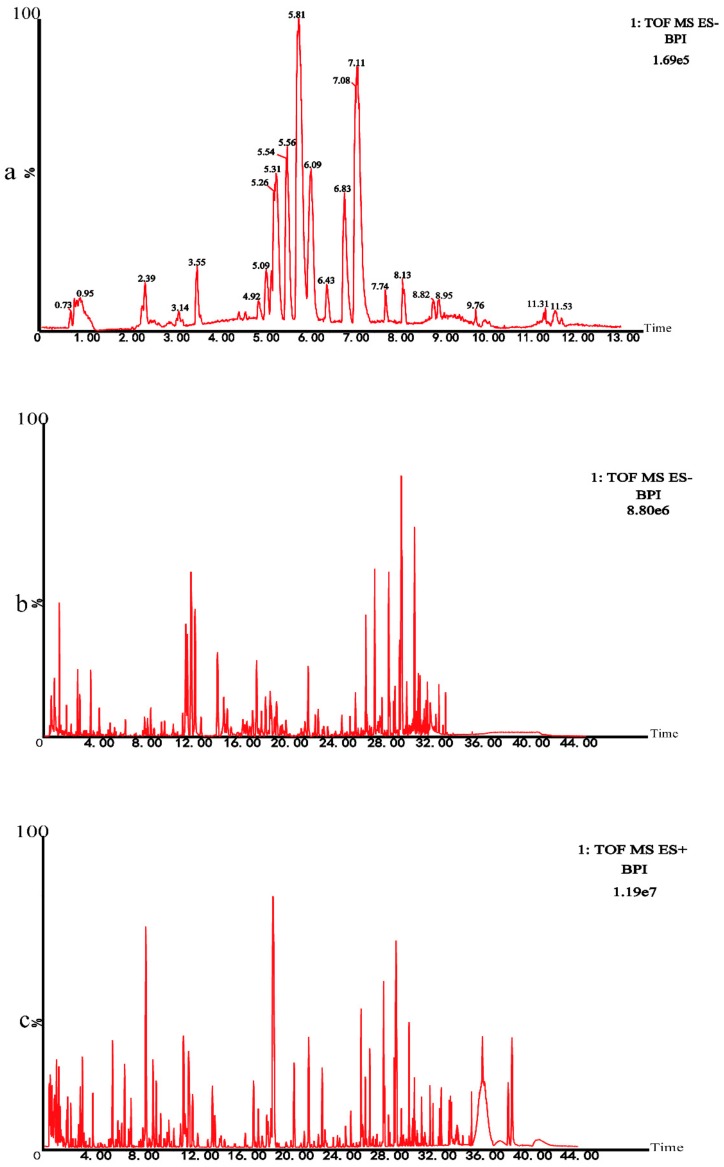
(**a**) The base peak intensity (BPI) chromatogram of plasma in the QC sample in negative mode was obtained based on the UHPLC–Q–TOF/MS platform. (**b**) The BPI chromatogram of chemical characterization in negative mode. (**c**) The BPI chromatogram of chemical characterization in positive mode.

Instrument precision experiment: A Quality control (QC) sample was injected 6 times consecutively. Twenty chromatographic peaks were selected randomly to calculate RSD values of the areas and retention time of these peaks. Method repeatability experiment: Six QC samples were prepared in parallel and continuous injection analysis was performed. Twenty chromatographic peaks were randomly selected to calculate RSD values of the areas and retention time of these peaks. Sample stability test: The same QC sample solution was taken and analyzed at 0, 6, 12, 18, and 24 h. The results of these analyses were presented in the Table 1. The results showed that the RSD of peak area was less than 15.0% and the RSD of retention time was less than 1.0%, indicating that the instrument precision, method repeatability, and sample stability were good. All results complied with the requirements. UHPLC-Q-TOF/MS technique was used to analyze the metabolomics of rats. In negative ion modes, the base peak intensity (BPI) diagram of QC samples of the subject was shown in Figure 3a. Subsequently, we conducted the chemical characterization test on Chinese medicine prescriptions by the same technology. The BPI chromatogram of chemical characterization in the negative mode was shown in Figure 3b. The BPI chromatogram of chemical characterization in the positive mode was shown in Figure 3c.

Based on the MS/MS results and related literature, 41 major chemical constituents were identified (Table 2).

### 2.4. Multivariate Data Analysis

**Figure 4:**
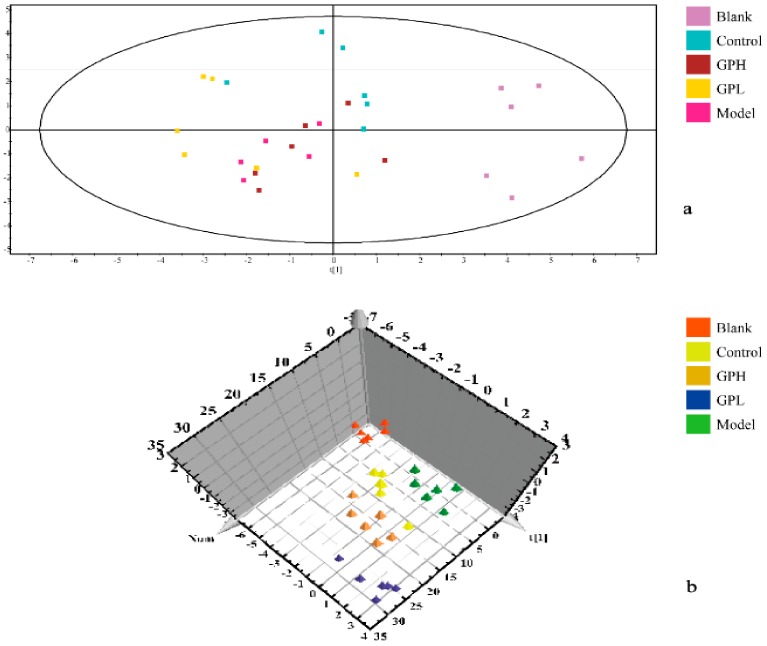
Results of multivariate statistical analysis. (**a**) principal component analysis (PCA) 2D scores plots of blank group, control group, and administration groups of Gout Party compared with model group. (**b**) partial least squares pattern recognition analysis (PLS–DA) 3D scores plots of blank group, control group, and administration groups of Gout Party compared with model group.

In the experiment, unsupervised PCA of the original data was first performed, and the most realistic metabolic differences between the reaction groups were obtained. As shown in Figure 4a, the separation was not ideal, and we performed PLS–DA analysis. The data were again subjected to supervised metabolomic analysis. The result was shown in Figure 4b. The blank and model groups were clearly separated in the three-dimensional figure, indicating that the modeling was successful, and the endogenous metabolites in the model group had been obviously changed. The results for the high-dose and control groups were moderate between those of the blank and model groups, and there was overlap between the high-dose and positive control groups, indicating that Gout Party had an effect on rats metabolism.

### 2.5. Optimization via ROC Curve Analysis

**Figure 5:**
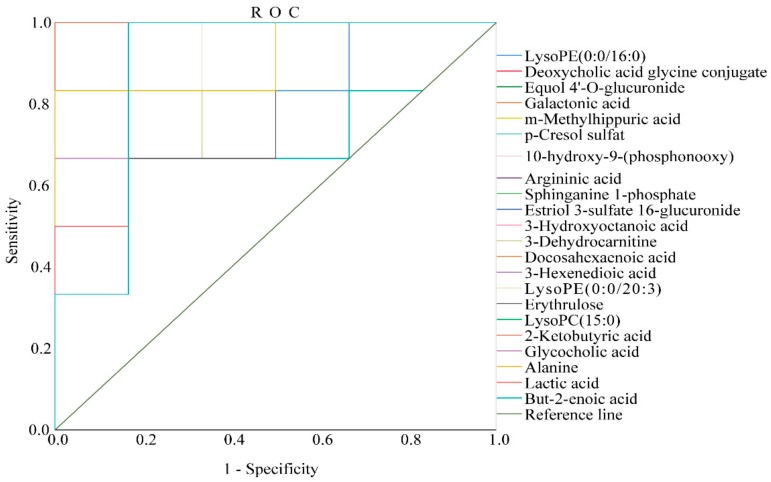
The receiver operating characteristic (ROC) curve to assess the predictive ability of the biomarkers of AGA.

In this experiment, we validated the diagnostic significance of the identified biomarkers via receiver operating characteristic (ROC) curve analysis. The ROC curve was based on a series of different two-category methods (demarcation value or decision threshold), with the true positive rate (sensitivity) as the ordinate and the false positive rate (1−specificity) as the abscissa. The area under the ROC curve (AUC) is usually used as an indicator of diagnostic efficiency. AUC > 0.85 indicates that the biomarker can be considered more diagnostic. In this experiment, the ROC curve was used to study the ability of these AGA biomarkers to diagnose AGA in rats. Of all the results, only two of the biomarkers had an area under the curve of less than 0.85, which were p–cresol sulfate 0.736 and erythrulose 0.778, respectively, and the remaining 20 substances were all greater than 0.88, demonstrating that the biomarkers had good diagnostic significance. (Please see Table S1 in Supplementary Materials for details.) The ROC curves of the AGA biomarkers were shown in Figure 5.

**Figure 6:**
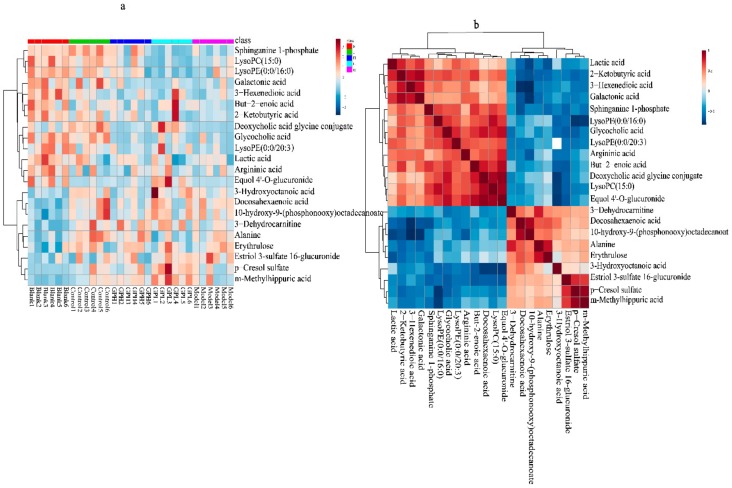
(**a**)Heatmap metabonomic data depicting the data structure of 22 biomarkers. The depth of the color represents the size of the variable. (**b**) Correlation metabonomic data depicting the data structure of 22 biomarkers. Red or blue represents the positive or negative correlation coefficients between metabolites, respectively.

To visually analyze the metabolite changes in vivo, we used hierarchical cluster analysis (i.e., heatmap) to analyze the biomarkers. The heatmap visually presented 22 distinct diagnostic biomarkers in all five groups. Figure 6a showed the variable information as the ordinate and the sample information as the abscissa, and the depth of the color represents the size of the variable. A closer bifurcation of the variable information in the vertical axis indicated greater similarity between substances, suggesting that they were probably derived from metabolites of the same substance. As shown in Figure 6, the changes in content in the blank and model groups were more obvious.

We also evaluated the relationships among the 22 biomarkers associated with AGA. As shown in Figure 6b, the differences between colors reflect the correlations between different metabolites. Overall, the 22 markers were closely related, estriol 3–sulfate 16–glucuronide, *p*–cresol sulfate (PCS), and m–methylhippuric acid had good correlations, the correlation coefficient as follow 0.74, 0.92243, 0.69, suggesting that these substances are closely related to the kidneys during the onset of AGA. Docosahexaenoic acid, but–2–enoic acid, LPC (15:0), and equol 4′–*O*–glucuronide had good correlations, the correlation coefficients are 0.85, 0.71, 0.904, respectively. Lactic acid, 2–ketobutyric acid, 3–hexenedioic acid, and galactonic acid had good correlations, the correlation coefficient as follow 0.76, 0.82, 0.72027, 0.89, 0.82, and they may play important roles in energy metabolism. Sphinganine 1–phosphate, LPE (0:0/16:0), glycocholic acid, and LPE (0:0/20:3) had good correlations, indicating that phospholipid metabolism was closely related to AGA. Docosahexaenoic acid and 10–hydroxy–9–(phosphonooxy)–octadecanoate had a good correlation, the correlation coefficient is 0.88, suggesting the potential importance of fatty acid metabolism. The correlation coefficient between alanine and erythrulose was 0.82, and thus, a critical role of amino acid metabolism can be speculated. The correlation matrix was available in the Supplementary Materials Table S2.

## 3. Discussion

**Figure 7:**
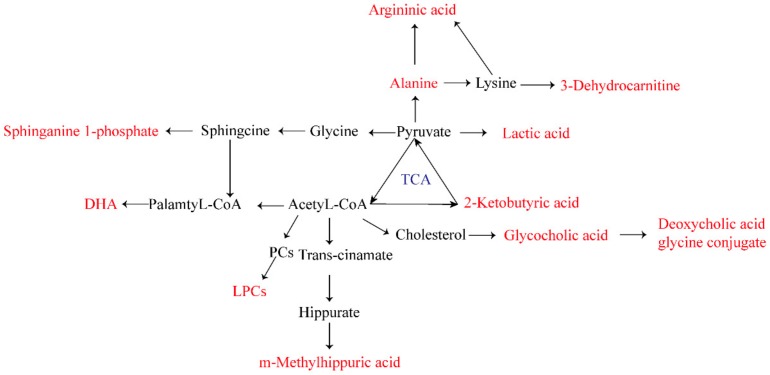
Biomarker pathway map of 22 biomarkers related to AGA.

In this experiment, we screened 22 biomarkers related to AGA (Table 3). Through the analysis of related metabolites, it was found that these metabolites were concentrated in fatty acid metabolism, bile acid metabolism, amino acid metabolism, and energy metabolism pathways. It is speculated that abnormalities of these four metabolic pathways were directly related to AGA. The biomarker pathway map is shown in Figure 7.

### 3.1. Amino Acid Metabolism

Twenty–two biomarkers associated with AGA were found and identified in the plasma of an SUC–induced AGA rats model. Three of these biomarkers are related to amino acid metabolism. Amino acids are most common in humans, and their metabolic processes and metabolites are involved in human immune and inflammatory responses [22]. Free amino acids in plasma are regulators of various metabolic pathways, and they play important roles in the biosynthesis and decomposition of various metabolites [23]. Alanine, a free amino acid in plasma, is one of the natural 20 amino acids that form human proteins. Mahbub et al. identified a significant positive correlation between alanine levels and gout. In the AGA model group, alanine levels were significantly higher than those in the blank group [24]. The primary clinical manifestation of gout is hyperuricemia, which is caused by abnormally high levels of uric acid [24,25]. 2-Ketobutyric acid is a substance that is involved in the metabolism of many amino acids (glycine, methionine, valine, leucine, serine, threonine, isoleucine). It can be converted into propionyl-CoA, and thus enter the citric acid cycle. Metabolism of 2–ketobutyric acid directly affects the normal metabolism of other amino acids, such as glycine. Glycine has a significant anti-inflammatory effect by inhibiting the activation of the nuclear transcription factor NF–κB, the degradation of IKB–α, and the production of IL–6 [26]. Plasma 2–ketobutyric acid level was decreased in model group rats, which affected the normal metabolism of glycine and led to a decreased anti–inflammatory effect. Arginine is also an important amino acid. Arginine metabolism is susceptible to increases of guanidine compound levels, resulting in its citrullination and conversion to citrulline. The citrullination process is common in pathological conditions such as rheumatoid arthritis and inflammatory joint diseases [27]. The decrease of arginine level in this experiment may be explained by the deepening of citrullination caused by AGA. In addition, it has been reported that the degradation of amino acids may be aggravated during inflammation [28].

### 3.2. Fatty Acid Metabolism

Fatty acid metabolism is an important and complex biochemical reaction in vivo involving the digestion, absorption, synthesis, and decomposition of fat in organisms with the help of various related enzymes. Through this process, fat is processed into substances needed by organisms to ensure the operation of normal physiological functions. Thus, fatty acid metabolism is of great significance for life activities. However, elevated levels of saturated fatty acids create conditions for the synthesis, processing, and expression of inflammatory factors, and severe cases can induce AGA [29,30]. But–2–enoic acid is a fatty acid formed by the action of fatty acid synthase on acetyl coenzyme A and a precursor of malonyl coenzyme A. 3–Hydroxyoctanoic acid is an intermediate in fatty acid biosynthesis. Both of these substances participate in fatty acid metabolism. In this experiment, the levels of but–2–enoic acid and 3–hydroxyoctanoic acid were lower in the model group than in the blank group, which may be related to fatty acid oxidation during the onset of AGA. In addition, fatty acid metabolism and fatty acid metabolites are associated with fatty acid oxidation processes, which increase inflammatory factor expression [31]. Docosahexaenoic acid (DHA) is essential for the growth and functional development of the brain in infants. However, many researches have shown that DHAis an omega–3 fatty acid metabolite that has potential anti-inflammatory activity in various inflammatory human diseases and beneficial effects on inflammatory diseases such as diabetes, asthma, and arthritis [31,32]. Abnormal metabolites contents indicated abnormalities in fatty acid metabolism or amino acid metabolism. Metabolic abnormalities led to inflammatory reactions, and levels of inflammatory factors IL-6 and IL-1β were elevated. 3–Hexenedioic acid is a normal human unsaturated dicarboxylic acid metabolite. Under the condition of inhibited fatty acid oxidation, 3–hexenedioic acid excretion is increased. The experimental results revealed that 3–hexenedioic acid level was decreased in the model group, which may be related to the anti-inflammatory self-regulation of the body.

Lipids are important nutrients in the human body. The energy needed to supply the body and the essential fatty acids needed by the body are also the components of human cell tissue. AGA episodes are often accompanied by disorders of lipid metabolism. Four lipid metabolites were identified in the experimental results, namely LPC (15:0), LPC (0:0/20:3), LPE (0:0/16:0), and sphinganine 1–phosphate. LPC is an endogenous phospholipid. As an important component of biofilm, LPC is closely related to the cell membrane, and it plays an important role in the regulation of human immunity and inflammation. The levels of LPC detected in this experiment were lower than those in the blank group [33,34,35]. This phenomenon suggested that the decrease of LPC content may be related to the occurrence of AGA.

10–Hydroxy–9–(phosphonooxy)–octadecanoate is a long-chain fatty acid that can be synthesized from octadecanoic acid (stearic acid). The deposition of SUCs in joints and synovium can lead to AGA. It has been suggested that the synergistic effect of stearic acid and sodium urate on inflammatory factors is positively correlated with the frequency of gout [36]. In addition, Joosten et al. demonstrated that stearic acid promotes the expression of inflammatory factors [30].

### 3.3. Bile Acid Metabolism

Bile acids are the main components of bile. Glycocholic acid is a bile acid, and studies have revealed that glycocholic acid has strong inhibitory effects on both acute and chronic inflammation [10]. Deoxycholic acid glycine conjugate is a secondary bile acid produced by the action of enzymes existing in microbial flora in the colonic environment. The conjugate at a certain concentration enhances the activity of fibrinolytic rheumatoid synovial fluid.

### 3.4. Energy Metabolism

Erythrulose is a highly reactive ketose that can rapidly saccharify and crosslink proteins, and it may be produced in diabetes. Studies indicated that uric acid is positively associated with elevated blood glucose [37,38]. The increase of erythrulose level in the model group may be directly related to AGA in the experiment. Lactic acid is a carboxylic acid that is continuously produced during metabolism and exercise. In the normal human body, all biochemical indicators are in dynamic balance, and the lactic acid level does not change significantly. However, in the experiment, lactic acid levels were significantly decreased in the AGA model rats, which may be attributable to accelerated gluconeogenesis, in which lactic acid is converted to glucose to meet the more urgent energy needs of diabetes mellitus [38]. Lactic acid has been considered an important biomarker in the pathogenesis of gout [39,40]. Estriol 3–sulfate 16–glucuronide is a natural human metabolite of estriol 3–sulfate produced by UDP–glucuronosyltransferase in the liver. Glucuronidation is used to facilitate the excretion of toxic substances, drugs, or other substances, and it does not produce energy. Increased blood glucose levels during AGA episodes may lead to increased glucuronidation and elevated levels of estriol 3–sulfate 16–glucuronide.

Carnitine has been considered a reliable biomarker for determining whether energy metabolism is abnormal. Carnitine transporters are responsible for the renal uptake and excretion of organic cations, and carnitine metabolism disorders can lead to abnormal transport, which in turn leads to decreased renal uric acid excretion [41,42,43].

We also identified PCS, m–methylhippuric acid, and equol 4′–*O*–glucuronide as potential biomarkers. Equol 4′–*O*–glucuronide is a polyphenol metabolite detected in biological fluids. Polyphenols have significant inhibitory effects on abnormal xanthine oxidase activity and elevated SUA levels during gout [37,44,45]. In a healthy state, microorganisms can regulate the immune system, prevent pathogenesis, and regulate the endogenous metabolism of carbohydrates and lipids, thus contributing to nutritional balance [46]. PCS is a microbial metabolite that is closely related to uremia and is the most common substance during uremia [47]. At excessive levels, PCS can increase renal fibrosis, decrease uric acid excretion by the kidneys, and stimulate uric acid accumulation, which can induce gout [48]. m–Methyl hippuric acid, also known as 3–methyl propionate, is an organic N–acyl–alpha amino acid synthesized from hippuric acid. Hippuric acid can affect the levels of uric acid and urea nitrogen.

## 4. Materials and Methods

### 4.1. Reagents and Materials

Saline (Shijiazhuang Four Medicine Co., Ltd., batch number 1704273203, China); colchicine, urea, and acetonitrile (HPLC Pure, Oceanpak, Sweden) were obtained from commercial sources.

All Chinese medicines are all purchased from Chinese medicine companies. Details are as follows:

*Aconiti Lateralis Radix Praeparaia* (hēi shun piàn; Tianjin Traditional Chinese Medicine Decoction Factory Co., Ltd., batch number 1801068–16, Sichuan); *Aconiti Radix Cocta* (chuān wū; Tianjin Traditional Chinese Medicine Decoction Factory Co., Ltd., batch No. G1803002–02, Sichuan), *Cremastrae Pseudobulbus Pleiones Pseudobulbus* (shān cí gū; Beijing Sheng shi long Pharmaceutical Co., Ltd., batch number 1804094, Guizhou), *Smilacis Glabrae Rhizoma* (tǔ fú líng; Anguo Sheng shan Pharmaceutical Co., Ltd., batch number 180301, Hunan), *Rehmanniae Radix* (dì huáng; Hebei North Pharmaceutical Co., Ltd., batch number 180701, Henan), and *Glycyrrhizae Radix et Rhizoma* (gān cǎo; Guangzhou Zhi xin Chinese Herbal Pieces Co., Ltd., Inner Mongolia).

The following devices were used in the study: Centrifuge (TDZ5–WS); toe volume measuring instrument (Anhui Zhenghua Biological Instrument Equipment Co., Ltd. ZH–ZZY); ultrasound cleaning machine (Ningbo Xinyi Biotechnology Co., Ltd. SB120D); vortex (Haimen Qilin Bell Instrument Manufacturing Co., Ltd.QL–091); Waters Acquity UPLC Liquid chromatograph (Waters, USA); Waters Xevo G2 Q–TOF mass spectrometer (Waters, USA); ACQUITY UPLC BEH C18 column (2.1 mm×100 mm×1.7 µm) (Waters, USA); and a Nikon Eclipse Ci Series microscope with a Nikon Digital Sight DS–FI2 camera (Japan).

### 4.2. Extraction of Gout Party

We weighed each Chinese herbal medicine (10 g) and decocted the medicine with a 10–fold volume of water for 40 min. The residue was filtered, and an 8–fold volume of water was added, followed by decoction for 40 min. The filtrate was combined twice and concentrated into an extract via decompression. This extract was used as an experimental drug. The weight was equal to 1 g/mL of the extract. Then, we accurately weighed the aforementioned Chinese medicines, namely hēi shun piàn 1 g, chuān wū 1 g, shān cí gū 2 g, tǔ fú líng 3 g, dì huáng 3 g, and gān cǎo 1 g. We placed hēi shun piàn and chuān wū in a round bottom flask and incubated the extract in a 10–fold volume of distilled water (20 mL) for 30 min. During this period, the remaining medicinal materials were soaked in a 10–fold volume of distilled water (170 mL). After the reflux, we poured the remaining medicinal materials and their soaking solution into a round bottom flask and refluxed the materials for 30 min. Then, the filtrate was stored, and an 8–fold volume of distilled water (152 mL) was added to the round bottom flask containing the dregs, which were refluxed again for 30 min. We combined the two reflux filtrates under reduced pressure filtration, centrifuged the filtrates at 5000 rpm for 10 min, and finally fixed the volume in a 250 mL volumetric flask for qualitative analysis via UHPLC–Q–TOF/MS.

Then, 6 mL of 1 mol/L NaOH solution were added to 194 mL of distilled water. After the mixture was boiled, 1 g of uric acid was added, and the pH was adjusted to 7.2 using 1 mol/L HCl. The resulting mixture was stirred continuously and stored in a refrigerator at 4 °C for 24 h. The clear liquid was placed in an oven at 70 °C for 2 h, and after removal, the dried product was ground into a powder to prepare SUCs.

Then, 250 mg of SUCs were added to 9 mL of normal saline and 1 mL of Tween–80 to prepare a sodium urate solution with a concentration of 25 mg/mL. We used an electronic balance to weigh 500 mg of SUCs, to which 2 mL of Tween–80 and sterile saline were added to a volume of 20 mL. The mixture was stirred with a magnetic stir bar until the crystal had completely dissolved to generate a 25 g/L sodium urate suspension. After high-pressure sterilization, the suspension was stored at 4 °C and shaken well before use [49].

Colchicine tablets were dissolved in 100 mL of ultrapure water to prepare a 0.04 g/L colchicine suspension that was sealed and stored at 4 °C for reserve.

### 4.3. Animals and Experimental Method

In total, 30 male Wistar rats weighing 180 ± 20 g were purchased from Speyer (Beijing) Biotechnology Co., Ltd. The rats were housed in cages and allowed free access to standard rat feed provided by Beijing Keao Co–operative Feed Co., Ltd. and pure water. New water bottles and fresh water were provided 2–3 times a week. After adaptive feeding, the rats were randomly divided into five groups (*n* = 6) as follows: Blank group, model group, colchicine group (control), Gout Party high-dose group (GPH), and Gout Party low-dose group (GPL).

The AGA model was created as described by Coderre. The injection site was the articular cavity between the median ankle joint and the tibia and fibula on the dorsal side of the right hindlimb of each rate. The ankle joint was placed at a right angle to fully expose the gap between the ankle joint and the tibia and fibula [50]. We injected 0.2 mL of the SUC suspension into the articular cavity directly from the gap at 45 °C. The dosage in each group is presented in the Table 4. All experimental procedures were conducted in accordance with Chinese national legislation and local guidelines. The entire study was performed with the permission and supervision of the Ethics Committee of Tianjin University of Traditional Chinese Medicine (20 Jun 2018) and the authorization number is SCXK2016–0006.

### 4.4. Sample Collection and Preparation

After seven days of administration, abdominal aorta blood was obtained under 10% chloral hydrate anesthesia (0.3 mL/kg) (before sample collection, all animals were fasted for 12 h, although water was allowed, to avoid the effects of food on the results). After the blood samples were taken from their abdominal aorta, the rats were euthanized by cervical dislocation. Tibial tissue was removed from each rat and fixed in 4% paraformaldehyde solution. The supernatant was centrifuged at 4 °C for 15 min at 3000 rpm/min. The supernatant was then centrifuged at 3500 rpm/min and 4 °C for 8 min to take the supernatant, and the resulting supernatant was stored in at −80 °C. Plasma samples removed from storage were thawed at room temperature, and 100 µL of serum were added to 300 µL of acetonitrile. Shocked it for 1 min and put it to ice bath to ultrasound for 10 min. We centrifuged the solution at 13,000 rpm/min for 15 min at 4 °C, and 200 µL of the supernatant were placed in a fresh vial.

After dissection, tibial tissue was fixed in 4% paraformaldehyde solution, dehydrated, and embedded in paraffin after taking the material. The pathological features of the tissues were examined using hematoxylin and eosin staining.

### 4.5. Metabolomic Analysis

This experiment used the UHPLC–Q–TOF/MS technique to perform metabolomic analysis of rat plasma. For detailed chromatographic and mass spectrometric conditions, please refer to the Table S3 in Supplementary Materials. We performed gradient elution as described in the Table 5. Via integrated analyses of data and multivariate statistical analyses, biomarkers related to AGA were identified, and their biological significance was explained to validate the UHPLC–Q–TOF/MS analysis. The precision, reproducibility, and stability of the specimens were determined using QC samples. Twenty of these samples were randomly selected to evaluate the relative standard deviation of precision and reproducibility. The specific methods of metabolomic data processing and analysis were as follows. First, the raw data collected at the workstation were extracted and exported using MassLynx (Version 4.1) software to obtain the retention time, *m/z* value, and peak area of the data. Then, the data were imported into SIMCA-P+12.0 software (Sweden Umetrics Company) for multivariate statistical analysis after 80% revision (Excel format). Initially, we used PCA for unsupervised data analysis, observed the clustering of each group of data, and removed outlier samples, and then we used PLS–DA for supervised data analysis. Based on the variable importance in projection (VIP) of the PLS–DA model, the differential candidate biomarkers were initially screened, and biomarkers with VIP > 1 were selected as potential biomarkers. Next, we used a *t*-test to screen for markers with significant differences (*p* < 0.05) as potential differential metabolic markers of AGA. To obtain an accurate molecular weight, we used the HMDB database to retrieve *m/z* values (http://www.hmdb.ca). Candidate biomarkers were identified via MS/MS analysis and literature searches. The binary logistic regression model of SPSS 25 software was used to calculate the ROC curves of AGA biomarkers in rats. Then, according to the contents of metabolites in each group, we generated heatmaps and correlation graphs using dedicated websites (https://www.metaboanalyst.ca/).

## 5. Conclusions

Based on the established non-targeted metabolomic analysis platform established in the laboratory, this study explored metabolic disorders caused by AGA and the effects of Gout Party on gout by analyzing different metabolites in rat plasma, and we explored the characteristics and mechanisms of Gout Party. The metabolomics results revealed that 14 of the 22 potential AGA biomarkers were altered after the administration of Gout Party, and these biomarkers were involved in processes including metabolic metabolism, fatty acid metabolism, and energy metabolism. The effective ingredients in Gout Party exert anti-inflammatory effects by inhibiting the secretion of the inflammatory factors IL–6 and IL–1β, thus playing a role in the treatment of AGA. It was demonstrated that Gout Party can reverse metabolic abnormalities in multiple pathways. Experimental studies have revealed that Gout Party can inhibit the growth of inflammatory cells induced by AGA and reverse abnormalities in plasma metabolite levels and metabolic pathways. Using metabolomics to determine the endogenous metabolites of AGA, we can explore the pathogenesis of AGA and provide a reliable basis for the prevention, detection, and diagnosis of the disease. However, non-targeted metabolomics analysis takes a long time, and a targeted metabolomics analysis, such as UHPLC-TQD-MS, is recommended in the future study. Secondly, we will be close to clinical research in the next study, we will do an in-depth study of its anti-inflammatory effects. With the continuous development of technology, metabolomics has become an effective means for Chinese medicine research and disease diagnosis and treatment, and it will facilitate the development of new models for the modernization of TCM.

## Figures and Tables

**Table 1:** The results of the experimental methodology.

Experiment Name	RSD (Peak Area)	RSD (Retention Time)
Instrument precision	<14.3%	<1.0%
Method repeatability	<13.7%	<1.0%
Sample stability	<14.8%	<1.0%

**Table 2:** Characterization of compounds in Gout Party

No.	tR (min)	*m/z* Obsd	*m/z* Calcd	Fromula	Identification	Parention	MS/MS
1	5.19	461.1674	461.1659	C_20_H_30_O_12_	Decaffeoyl-verbascoside	[M–H]^−^	161.04;135.04
2	5.86	486.2701	486.2703	C_24_H_39_NO_9_	Mesaconine	[M+H]^+^	468.25;454.24
3	6.17	387.1321	387.1291	C_17_H_24_O_10_	Genipin	[M–H]^−^	225.07
4	6.64	454.2778	454.2805	C_24_H_39_NO_7_	Fuziline	[M+H]^+^	436.28;404.28
5	6.86	358.2365	358.2382	C_22_H_31_NO_3_	Songorine	[M+H]^+^	340.23
6	7.33	500.2831	500.286	C_25_H_41_NO_9_	Aconine	[M+H]^+^	482.27;468.25
7	8.03	523.1666	523.1663	C_21_H_32_O_15_	Miltide	[M–H]^−^	463.14;343.10
8	8.58	470.2725	470.2754	C_24_H_39_NO_8_	Hypaconine	[M+H]^+^	438.24;406.22
9	8.76	454.2769	454.2805	C_24_H_39_NO_7_	Delectinine	[M+H]^+^	436.26;404.24
10	9.26	438.2823	438.2856	C_24_H_39_NO_6_	Neoline	[M+H]^+^	420.27;388.24
11	9.64	468.2935	468.2961	C_25_H_41_NO_7_	Browniine	[M+H]^+^	450.28;436.26
12	9.94	468.2935	468.2961	C_25_H_41_NO_7_	Lycoctonine	[M+H]^+^	362.29; 393.30
13	10.59	422.2890	422.2906	C_24_H_39_NO_5_	Talatizamine	[M+H]^+^	390.26;372.25
14	12.07	452.3019	452.3012	C_25_H_41_NO_6_	Chasmanine	[M+H]^+^	420.27;388.24
15	14.26	606.2914	606.2914	C_31_H_43_NO_11_	10-OH-Benzoylmesaco-nine	[M+H]^+^	588.27;574.26;556.25
16	15.99	623.1956	623.1976	C_29_H_36_O_15_	Acteoside	[M–H]^−^	461.16 161.02
17	17.44	576.2809	576.2809	C_30_H_41_NO_10_	Unknown	[M+H]^+^	558.27;544.25;526.24
18	19.01	544.2915	544.2910	C_30_H_41_NO_8_	Gadenine	[M+H]^+^	512.26;494.25;484.23
19	21.12	604.3154	604.3122	C_32_H_45_NO_10_	Benzoylaconitine	[M+H]^+^	588.31
20	22.26	572.2848	572.2860	C_31_H_41_NO_9_	Pyromesaconitine	[M+H]^+^	554.27;540.25;522.24
21	22.35	574.3044	574.3016	C_31_H_43_NO_9_	BenzoylhypaconineS	[M+H]^+^	542.27;510.24
22	22.59	516.2947	516.2961	C_29_H_41_NO_7_	Unknown	[M+H]^+^	442.25;414.26
23	23.31	616.3135	616.3122	C_33_H_45_NO_10_	Pyrojesaconitine	[M+H]^+^	598.30;584.28;566.27
24	23.78	558.3096	558.3067	C_31_H_43_NO_8_	Deoxybenzoylhypaconitine	[M+H]^+^	526.27;508.27
25	23.88	648.3031	648.3020	C_33_H_45_NO_12_	Beiwutine	[M+H]^+^	588.29
26	24.95	556.2905	556.2910	C_31_H_41_NO_8_	Pyrohypaconitine	[M+H]^+^	524.26;492.23
27	25.25	586.3019	586.3016	C_32_H_43_NO_9_	Pyroaconitine	[M+H]^+^	568.29;554.27;472.21
28	25.64	632.3107	632.3071	C_33_H_45_NO_11_	16-O-demethylaconitine	[M+H]^+^	572.28;540.25;508.22
29	25.64	632.3107	632.3071	C_33_H_45_NO_11_	Mesaconitine	[M+H]^+^	572.30;540.30
30	27.00	570.3082	570.3067	C_32_H_43_NO_8_	Pyrodeoxyaconitine	[M+H]^+^	538.27;506.25;478.22
31	27.11	616.3145	616.3122	C_33_H_45_NO_10_	HypaconitineS	[M+H]^+^	584.28;556.28;524.26
32	27.66	628.3131	628.3122	C_34_H_45_NO_10_	Anhydroaconitine	[M+H]^+^	568.28;536.26;508.26
33	28.33	700.2738	700.2758	C_39_H_41_NO_11_	Trifoliolasine E	[M+H]^+^	640.25;578.23
34	28.55	630.3281	630.3278	C_34_H_47_NO_10_	Deoxyaconitine	[M+H]^+^	598.30;570.30;538.27
35	28.74	505.3532	505.3529	C_30_H_48_O_6_	16-Oxo-alisol A	[M+H]^+^	505.33;487.34
36	29.32	529.3539	529.3529	C_32_H_48_O_6_	Alisol C-23-acetate	[M+H]^+^	551.33;511.34
37	30.92	455.3502	455.3525	C_30_H_46_O_3_	Alisol I	[M+H]^+^	477.33;543.33
38	31.67	487.3411	487.3423	C_30_H_46_O_5_	Alisol C	[M+H]^+^	509.33;469.32
39	31.71	489.3563	489.3580	C_30_H_48_O_5_	Alisol F	[M+H]^+^	511.33;471.34
40	32.51	471.3463	471.3474	C_30_H_46_O_4_	Alisol H	[M+H]^+^	493.32;453.33
41	33.98	515.3711	515.3736	C_32_H_50_O_5_	Alisol B-23-acetate	[M+H]^+^	537.35;497.35

**Table 3:** The information of the AGA biomarkers.

No.	tR(min)	*m/z* Obsd	*m/z* Calcd	MS/MS	PPM	Metabolites	Formula	Content Variance			
								Model	High	Low	Control
1	0.79	101.0234	101.0239	53.00, 83.01	−4.95	2–Ketobutyric acid	C_4_H_6_O_3_	↓**	↑	↑	↑
2	0.79	143.0344	143.0344	59.01 81.03	0.00	3–Hexenedioic acid	C_6_H_8_O_4_	↓**	↑	↑	↑
3	0.79	85.0288	85.0290	67.01 68.99	–2.35	But–2–enoic acid	C_4_H_6_O_2_	↓**	↓	↑	↑
4	0.81	174.0878	174.0879	100.08 112.09	−0.57	Argininic acid	C_6_H_13_N_3_O_3_	↓**	↑	↓	↑
5	0.81	195.0487	195.0505	89.02	−9.23	Galactonic acid	C_6_H_12_O_7_	↓**	↑	↑	↑
6	0.84	88.0394	88.0399	88.04	−5.68	Alanine	C_3_H_7_NO_2_	↑**	↑	↓	↑
7	0.91	119.0342	119.0344	87.01 89.02 101.02	−1.68	Erythrulose	C_4_H_8_O_4_	↑*	↓	↓	↓
8	0.92	89.0235	89.0239	71.01 73.00 89.02	−4.49	Lactic acid	C_3_H_6_O_3_	↓**	↑	↓	↑
9	2.36	158.0814	158.0817	59.01 84.99 101.02	–1.90	3–Dehydrocarnitine	C_7_H_13_NO_3_	↑**	↓	↑	↓
10	2.41	192.0658	192.0661	65.04 74.02 77.04	−1.56	m–Methylhippuric acid	C_10_H_11_NO_3_	↑*	↑	↑#	↑
11	2.52	417.1171	417.1186	103.04 121.03	−3.60	Equol 4’–O–glucuronide	C_21_H_22_O_9_	↓**	↓	↑##	↓
12	2.88	159.1016	159.1021	59.01 71.03 87.01	−3.14	3–Hydroxyoctanoic acid	C_8_H_16_O_3_	↑*	↑	↑	↑
13	2.95	187.0059	187.0065	51.02 75.02 77.04	−3.21	p–Cresol sulfate	C_7_H_8_O_4_S	↑*	↓	↑#	↑
14	3.08	448.3050	448.3063	74.02 116.04	−2.90	Deoxycholic acid glycine conjugate	C_26_H_43_NO_5_	↓*	↓	↓##	↓##
15	3.09	465.3028	465.3039	116.04 373.27	−2.36	Glycocholic acid	C_27_H_46_O_4_S	↓**	↓	↑#	↑#
16	4.78	380.2552	380.2566	78.96 96.97	−3.68	Sphinganine 1–phosphate	C_18_H_40_NO_5_P	↓**	↓	↓	↓##
17	5.69	502.2945	502.2934	78.96 140.01 153.00	2.19	LPE (0:0/20:3)	C_25_H_46_NO_7_P	↓**	↑	↑	↑
18	5.76	452.2775	452.2777	98.99 140.01 196.04	–0.44	LPE (0:0/16:0)	C_21_H_44_NO_7_P	↓*	↑	↓	↑
19	7.04	480.3089	480.3090	78.96 196.04 265.25	−0.21	LPC (15:0)	C_23_H_48_NO_7_P	↓**	↑	↓#	↑##
20	7.75	543.1513	543.1536	59.01 87.01 89.02	−4.24	Estriol 3–sulfate 16–glucuronide	C2_4_H_32_O_12_S	↑*	↓	↑	↑
21	7.76	395.2190	395.2199	62.96 96.97 113.13	−2.28	10–Hydroxy–9–(phosphonooxy)octadecanoate	C_18_H_37_O_7_P	↑**	↓	↑	↑
22	7.76	327.2320	327.2324	127.08 161.13 163.15	–1.22	Docosahexaenoic acid	C_22_H_32_O_2_	↑**	↓	↓	↑

↑**: Extremely significantly increased compared with blank group (*p* < 0.01); ↓**:Extremely significantly decreased compared with blank group (*p* < 0.01); ↑*: Significantly increased compared with blank group (*p* < 0.05); ↓*: Significantly decreased compared with blank group (*p* < 0.05); ↑##:Extremely significantly increased compared with model group (*p* < 0.01); ↓#: Significantly decreased compared with model group (*p* < 0.05); ↑#: Significantly increased compared with model group (*p* < 0.05); ↓##: Extremely significantly decreased compared with model group.

**Table 4:** Experimental groups and dosage of each group.

Groups	Administration	Dosage	Mode of Administration	Time	*N*
Blank	Saline	15 mL/kg	Gavage	7 days	6
Model	Saline	15 mL/kg	Gavage	7 days	6
Control	Colchicine	0.8 mg/kg	Gavage	7 days	6
GPH	Gout Party	20 g/kg	Gavage	7 days	6
GPL	Gout Party	1 0g/kg	Gavage	7 days	6

**Table 5:** UHPLC–Q–TOF/MS gradient elution method.

T (min)	Phase A (0.1% Formic Acid in Water)	Phase B (0.1% Formic Acid Inacetonitrile)
0	99.0	1.0
0.5	99.0	1.0
2	50.0	50.0
9	1.0	99.0
10	1.0	99.0
11	99.0	1.0
13	99.0	1.0

## Supplementary Materials

Supplementary Materials can be found at https://www.mdpi.com/1422-0067/20/22/5753/s1.

## Abbreviations

GA	Gouty arthritis
AGA	Acute gouty arthritis
UPLC–Q–TOF/MS	Ultra-high-performance liquid chromatography–tandem quadrupole/time-of-flight mass spectrometry
PCA	Principal component analysis
PLS-DA	partial least square discrimination analysis
SUCs	Sodium urate crystals
SUA	Serum uric acid
TCM	Traditional Chinese medicine
ELISA	Enzyme–linked immunosorbent assay
QC	Quality control
BPI	base peak intensity
ROC	receiver operating characteristic
PCS	*p*–cresol sulfate
GPH	Gout Party high–dose group
GPL	Gout Party low–dose group
VIP	Variable importance in projection
