# Supplementary material for: Plasma Metabolic Profiling Analysis of Gout Party on Acute Gout Arthritis Rats Based on UHPLC–Q–TOF/MS Combined with Multivariate Statistical Analysis
Source: Int J Mol Sci. 2019 Nov 15;20(22):5753. doi: 10.3390/ijms20225753 (PMC6888674; doi:10.3390/ijms20225753)
Supplement: Supplementary file 1 [file ijms-20-05753-s001.pdf]

Table S1. The ROC AUC.

	AUC
LPE(0:0/16:0)	0.889
Deoxycholic acid glycine conjugate	0.889
Equol 4'–O–glucuronide	1.000
Glycocholic acid	0.917
m–Methylhippuric acid	0.917
p–Cresol sulfate	0.736
10–Hydroxy–9–(phosphonooxy)octadecanoate	0.972
Argininic acid	0.889
Sphinganine 1–phosphate	1.000
Estriol 3–sulfate 16–glucuronide	0.917
3–Hydroxyoctanoic acid	0.861
3–Dehydrocarnitine	0.889
Docosahexaenoic acid	1.000
3–Hexenedioic acid	0.944
LPE(0:0/20:3)	0.944
Erythrulose	0.778
LPC(15:0)	0.972
2–Ketobutyric acid	1.000
Glycocholic acid	0.889
Alanine	0.917
Lactic acid	0.917
LPE(0:0/16:0)	0.889

Chromatographic and mass spectrometric conditions

HPLC analysis was performed using a Waters UHPLC-Q-TOF/MS system (Waters, USA). Plasma samples (5 mL) were injected into an ACQUITY UPLC BEH C18 column (2.1×100 mm×1.7 μm, Waters), The column temperature was 40°C, and the flow rate was 0.3 mL min^{-1}. Q-TOF/MS was equipped with electrospray ionisation in negative mode. The MS parameters were as follows: drying gas temperature, 450°C; drying gas flow, 10mL /min; desolvation gas flow, 800 L/h; capillary voltage, 2.5kV; Ionization source temperature, 100℃; reference ions [M-H]$^-$=554.2615; data acquisition range, m/z 50-1000Da.

Table S2. The correlation matrix of 22 Biomarkers

	Lactic acid	2-Ketobutyric acid	3-Hexenedioic acid	Galactonic acid	Sphinganine 1–phosphate	LysoPE(0:0/16:0)	Glycocholic acid	LysoPE(0:0/20:3)	Argininic acid	But-2-enoic acid	Deoxycholic acid glycine conjugate	LysoPC(15:0)	Equol 4'–O–glucuronide	3-Dehydrocarnitine	Docosahexaenoic acid	10–hydroxy–9–(phosphonooxy)octadecanoate	Alanine	Erythrulose	3-Hydroxyoctanoic	Estriol 3–sulfate 16–glucuronide	p-Cresol sulfate	m-Methylhippuric acid
Lactic acid	1	0.76006	0.60195	0.72027	0.42614	0.24551	0.435	0.62007	0.52683	0.47303	0.1727	0.2287	0.31871	-0.31238	-0.61055	-0.73166	-0.49468	-0.67904	-0.10204	-0.45991	-0.43661	-0.33435
2-Ketobutyric acid	0.76006	1	0.81768	0.89191	0.62667	0.61978	0.66733	0.58354	0.58128	0.71246	0.42247	0.53664	0.5976	-0.36412	-0.65499	-0.69317	-0.62601	-0.60503	-0.48571	-0.52439	-0.62021	-0.55234
3-Hexenedioic acid	0.60195	0.81768	1	0.81701	0.49742	0.56108	0.43689	0.44062	0.57246	0.35551	0.24776	0.43123	0.3943	-0.42436	-0.79982	-0.82513	-0.53913	-0.50894	-0.44627	-0.34001	-0.55416	-0.55277
Galactonic acid	0.72027	0.89191	0.81701	1	0.42265	0.4062	0.38696	0.39454	0.60067	0.58036	0.26351	0.33095	0.42436	-0.33572	-0.67072	-0.78913	-0.60149	-0.48179	-0.33997	-0.49435	-0.52588	-0.46075
Sphingani	0.	0.62	0.49	0.4	1	0.679	0.62	0.6310	0.3	0.	0.56	0.69	0.61	-	-	-0.48426	-	-	-	-	-	-

ne 1–phosphate	42614	667	742	2265		84	778	5	1237	71996	69	15	316	0.36994	0.68542		0.490038	0.59467	0.50049	0.63236	0.45103	0.5714
LysoPE(0:0/16:0)	0.24551	0.61978	0.56108	0.4062	0.67984	1	0.7081	0.63501	0.4414	0.62058	0.65911	0.8391	0.70579	-0.28395	-0.43903	-0.2654	-0.18087	-0.14222	-0.62573	-0.58924	-0.78215	-0.76161
Glycocholic acid	0.435	0.66733	0.43689	0.38696	0.62778	0.7081	1	0.74864	0.60536	0.7199	0.81189	0.76019	0.84166	-0.56609	-0.35222	-0.24902	-0.44352	-0.31261	-0.60294	-0.64718	-0.64324	-0.50612
LysoPE(0:0/20:3)	0.62007	0.58354	0.44062	0.39454	0.63105	0.63501	0.74864	1	0.64789	0.60684	0.57166	0.59167	0.64399	-0.50893	-0.52191	-0.4969	-0.31105	-0.3243	-0.51686	-0.56775	-0.55262	-0.50154
Argininic acid	0.52683	0.58128	0.57246	0.60067	0.31237	0.4414	0.60536	0.64789	1	0.54255	0.63389	0.63256	0.7707	-0.47101	-0.47211	-0.6604	-0.22328	-0.057392	-0.56218	-0.56929	-0.39598	-0.24036
But-2-enoic acid	0.47303	0.71246	0.35551	0.58036	0.71996	0.62058	0.7199	0.60684	0.54255	1	0.78914	0.71211	0.85061	-0.22021	-0.46287	-0.38922	-0.32491	-0.24632	-0.64797	-0.65706	-0.42824	-0.31157
Deoxycholic acid glycine conjugate	0.1727	0.42247	0.24776	0.26351	0.5669	0.65911	0.81189	0.57166	0.63389	0.78914	1	0.88072	0.94122	-0.47142	-0.3578	-0.19718	-0.2082	0.0064201	-0.76387	-0.63446	-0.4135	-0.25572

																1				1	
LysoPC(15:0)	0.2287	0.53664	0.43123	0.33095	0.6915	0.8391	0.76019	0.59167	0.63256	0.71211	0.88072	1	0.90448	-0.42246	-0.46184	-0.32103	-0.22163	-0.14652	-0.75488	-0.64629	-0.51726	-0.46464
Equol 4'–O–glucuronide	0.31871	0.5976	0.3943	0.42436	0.61316	0.70579	0.84166	0.64399	0.7707	0.85061	0.94122	0.90448	1	-0.37502	-0.39163	-0.3524	-0.20182	-0.054462	-0.77329	-0.66208	-0.45246	-0.30373
3-Dehydrocarnitine	-0.31238	-0.36412	-0.42436	-0.33572	-0.36994	-0.28395	-0.56609	-0.50893	-0.47101	-0.22021	-0.47142	-0.42246	-0.37502	1	0.5457	0.41411	0.77368	0.4698	0.42224	0.40362	0.3439	0.35512
Docosahexaenoic acid	-0.61055	-0.65499	-0.79982	-0.67072	-0.68542	-0.43903	-0.35222	-0.52191	-0.47211	-0.46287	-0.3578	-0.46184	-0.39163	0.5457	1	0.88471	0.61073	0.63126	0.54096	0.3365	0.2884	0.34854
10–hydroxy–9–(phosphonooxy)octadecanoate	-0.73166	-0.69317	-0.82513	-0.78913	-0.48426	-0.2654	-0.24902	-0.4969	-0.6604	-0.38922	-0.19718	-0.32103	-0.3524	0.41411	0.88471	1	0.50878	0.54689	0.40371	0.30975	0.21547	0.23862
Alanine	-0.49468	-0.62601	-0.53913	-0.60149	-0.49038	-0.18087	-0.44352	-0.31105	-0.22328	-0.32491	-0.20821	-0.22163	-0.20182	0.77368	0.61073	0.50878	1	0.81552	0.25199	0.3397	0.30608	0.38127

Erythrulose	-0.67904	-0.60503	-0.50894	-0.48179	-0.59467	-0.14222	-0.31261	-0.3243	-0.057392	-0.24632	0.0064201	-0.14652	-0.054462	0.4698	0.63126	0.54689	0.81552	1	0.029764	0.26736	0.23425	0.35927
3-Hydroxyoctanoic	-0.10204	-0.48571	-0.44627	-0.33997	-0.50049	-0.62573	-0.60294	-0.51686	-0.56218	-0.64797	-0.76387	-0.75488	-0.77329	0.42224	0.54096	0.40371	0.25199	0.029764	1	0.17482	0.15509	0.089025
Estriol 3–sulfate 16–glucuronide	-0.45991	-0.52439	-0.34001	-0.49435	-0.63236	-0.58924	-0.64718	-0.56775	-0.56929	-0.65706	-0.63446	-0.64629	-0.66208	0.40362	0.3365	0.30975	0.3397	0.26736	0.17482	1	0.74181	0.69373
p-Cresol sulfate	-0.43661	-0.62021	-0.55416	-0.52588	-0.45103	-0.78215	-0.64324	-0.55262	-0.39598	-0.42824	-0.41351	-0.51726	-0.45246	0.3439	0.2884	0.21547	0.30608	0.23425	0.15509	0.74181	1	0.92243
m-Methylhippuric acid	-0.33435	-0.55234	-0.55277	-0.46075	-0.5714	-0.76161	-0.50612	-0.50154	-0.24036	-0.31157	-0.25572	-0.46464	-0.30373	0.35512	0.34854	0.23862	0.38127	0.35927	0.089025	0.69373	0.92243	1

Table S3. UHPLC–Q–TOF/MS gradient elution method

T(min)	Phase A (0.1% formic acid) in water	Phase B (0.1% formic acid) in acetonitrile
0	98	2
5	94	6
10	87	13
15	85	15
20	80	20
25	72	28
30	60	40
35	15	85
40	15	85
42	98	2
45	98	2

spectrometric conditions:

The MS parameters were as follows: drying gas temperature, 450°C; drying gas flow, 10mL /min; desolvation gas flow, 800 L/h; capillary voltage, 2.5kV; Ionization source temperature, 100℃.
